# Attitude and knowledge of intensive care staff concerning donation in Hungary: it is the first step to change

**DOI:** 10.1186/cc12449

**Published:** 2013-03-19

**Authors:** A Smudla, S Mihály, J Fazakas

**Affiliations:** 1 Semmelweis University, Budapest, Hungary; 2 Hungarian National Blood Transfusion Service, Budapest, Hungary

## Introduction

In Hungary, despite the high level of social support, the number of organ recoveries from deceased donors has not changed significantly. The donation activity shows a positive relationship with the level of education of staff in ICUs as well as with their attitude towards transplantation. The aim of this cross-sectional study is to estimate the attitude and knowledge of intensive care specialists and nurses as regard donation and transplantation.

## Methods

The self-completed questionnaire that consisted of 20 items was completed at the Congress of Hungarian Society of Anesthesiology and Intensive Therapy in 2011. Besides the epidemiological data, the intensive care specialists (*n *= 179) and nurses (*n *= 103) were asked about donation activity, participation in an organ donation course, self-reported knowledge of joining Eurotransplant, donor management, legislation, and transplantation. The data were analyzed by SPSS 17.0.

## Results

A total of 53.6% of physicians and 16.7% of nurses attended an earlier organ donation course (*P *0.01). The average age of those who participated in training was significantly higher among doctors (*P *0.01). Fifty-nine percent of doctors and 65.1% of nurses did not even want to participate in such training. Donation activity was higher among staff who joined training (*P *0.01). Independently from accepting the presumed consent legislation (91.1%), 66% of physicians agreed with the hospital practice that requests the adult donor's relatives to consent to organ recovery. This standpoint did not depend on donation activity, participation in an organ donation course, opinion about legislation and the nature of staff. A total 95.4% of participants consented to their organ retrieval after death. The staff who participated in an organ donation course had more knowledge regarding the law and ethics of donation (*P *0.01), donor management (*P *0.01), living and deceased donor transplantation (*P *0.01) and joining Eurotransplant (*P *0.01). Older professionals had more information about all fields (*P *0.01). Nurses had less knowledge concerning donor management (*P *0.01), law and ethics (*P *0.01) and deceased donor transplantation (*P *0.01) than doctors.

## Conclusion

Education about organ donation needs to be part of specialist training of intensive care staff, and refresher courses every fifth year as well. The course should include knowledge regarding brain death, donor management and communication with family. This is the first step to improve the number of transplantations.
